# Phylogenomics insights into order and families of *Lysobacterales*

**DOI:** 10.1099/acmi.0.000015

**Published:** 2019-04-17

**Authors:** Sanjeet Kumar, Kanika Bansal, Prashant P. Patil, Prabhu B. Patil

**Affiliations:** 1 Bacterial Genomics and Evolution Laboratory, CSIR-Institute of Microbial Technology, Chandigarh, India; ^†^​ Present address: Department of Archaeogenetics, Max Planck Institute for the Science of Human History, Jena, Germany; ^‡^​ Present address: Department of Microbiology, School of Medicine, University of Washington, Seattle, WA, USA

**Keywords:** type species, type strains, phylogenomics, order, taxogenomics, *Lysobacterales*

## Abstract

Order *Lysobacterales* (earlier known *Xanthomonadales *) is a taxonomically complex group of a large number of gamma-proteobacteria classified in two different families, namely *Lysobacteraceae* and *Rhodanobacteraceae *. Current taxonomy is largely based on classical approaches and is devoid of whole-genome information-based analysis. In the present study, we have taken all classified and poorly described species belonging to the order *Lysobacterales* to perform a phylogenetic analysis based on the 16 S rRNA sequence. Moreover, to obtain robust phylogeny, we have generated whole-genome sequencing data of six type species namely *Metallibacterium scheffleri , Panacagrimonas perspica , Thermomonas haemolytica , Fulvimonas soli , Pseudofulvimonas gallinarii* and *Rhodanobacter lindaniclasticus* of the families *Lysobacteraceae* and *Rhodanobacteraceae *. Interestingly, whole-genome-based phylogenetic analysis revealed unusual positioning of the type species *Pseudofulvimonas *, *Panacagrimonas , Metallibacterium* and *Aquimonas* at family level. Whole-genome-based phylogeny involving 92 type strains resolved the taxonomic positioning by reshuffling the genus across families *Lysobacteraceae* and *Rhodanobacteraceae *. The present study reveals the need and scope for genome-based phylogenetic and comparative studies in order to address relationships of genera and species of order *Lysobacterales *.

## Impact Statement

Species of order *Lysobacterales* have undergone several reclassifications, until today the taxonomy position of species within the order is largely devoid of whole-genome information. The order *Lysobacterales* includes more than 200 species with great biotechnological potential. Current phylogeny is based on 16S rRNA gene phylogeny, conserved signature indels (CSIs) and classical approaches, such as morphological, biochemical or low-resolution molecular biology methods. Major limitations of these are not including all species of the order and are based on single or few conserved gene sequences. With the revolution in genome sequencing, whole genome can be utilized in a more profound way to investigate the phylogeny. Hence, this study attempts to achieve robust taxonomy and phylogeny of the order by sequencing type strains and including them up to the whole-genome level resolution. Our basic phylogenomic analysis revealed the existence of novel families within the order. In addition, reshuffling of the genera within the order across families is evident based on whole-genome sequence data. Whole-genome information of the type strain of the genus with unary species can serve as a reference and standard to compare later identified species of the respective genera.

## Data Summary

1. Draft genome assemblies of six type species of order *Lysobacterales* have been deposited to GenBank and the accession number of each has been provided in Table 1.
2. Phylogenetic tree file in Newick format (.nwk), constructed using the maximum-likelihood method of the 16S rRNA gene sequence of the 206 type strains of order Lysobacterales (also includes the strains that were previously removed).
3. Phylogenetic tree file in Newick file (.nwk), constructed using the 400 most conserved genes from the whole-genome data using PhyloPhlAn.
4. Data file (G-I_OrthoANI.xlxs) used to generate the heat map of orthoANI values of all subgroups (G-I) found to have an unusual grouping in the phylogenetic tree construction using the >400 most conserved genes.
5. Data file (G-II-OrthoANI.xlsx) used to generate the heat map of orthoANI values of all subgroups (G-II) found to have an unusual grouping in the phylogenetic tree construction using the >400 most conserved genes.
6. Data file (G-I_POCP.xlsx) used to generate the heat map of the percentage of conserved protein (POCP) values of the unusual grouping (G-I) obtained from the phylogenetic construction.
7. Data file (G2-POCP.xlxs) used to generate the heat map of the percentage of conserved protein (POCP) values of the unusual grouping (G-I) obtained from the phylogenetic construction.

**Table 1.** Genome feature of type species of family *Lysobacteraceae* and *Rhodanobacteraceae* under study. Genome sequences of type species reported in the present study are highlighted in bold

Strain name	Genome (Mb)	Completeness (%)	Contamination (%)	Coverage	#Contigs	N50 (bp)	GC (%)	#CDS	rRNA + tRNA	Accession no.
* Arenimonas donghaensis* DSM 18148 (T)	2.98	99.37	0.72	332.4x	51	159 562	68.8	2710	4+45	AVCJ01000000
* Luteimonas mephitis* DSM 12574 (T)	3.33	99.48	0.36	–	20	471 857	68.5	3011	3+45	AULN01000000
* Lysobacter enzymogenes* ATCC 29487 (T)	6.26	99.8	2.17	208x	68	155 039	69.3	5029	5+54	FNOG01000000
*** Metallibacterium scheffleri* DSM 24874** (T)	**3.59**	**99.5**	**3.84**	**318x**	**119**	72 249	**65.9**	3242	**3+** **45**	**MWQO00000000**
*** Panacagrimonas perspica* DSM 26377** (T)	**5.39**	**98.82**	**1.81**	**158.5x**	**85**	**222 295**	**66**	4848	**3+** **47**	**MWIN00000000**
* Silanimonas lenta* DSM 16282 (T)	2.64	99.66	0.04	–	24	279 398	71.1	2395	10+49	AUBD01000000
* Stenotrophomonas maltophilia* ATCC13637 (T)	4.98	100	0.34	417x	1	–	66.1	4588	7+67	CP008838
*** **Thermomonas haemolytica*** LMG 19653** (T)	**2.54**	**98.93**	**1.48**	**250x**	**105**	70 437	**70**	2373	**3+** **46**	**MSZW00000000**
* Xanthomonas campestris* ATCC 33913 (T)	5.07	99.64	0	–	1	–	65.1	4179	6+53	AE008922
* Xylella fastidiosa* ATCC 35871 (T)	2.41	99.64	0	–	66	102 517	51.7	2227	7+49	AUAJ01000000
* Aquimonas voraii* DSM 16957 (T)	4.42	99.66	0.93	220x	41	288 128	68.5	3569	6+47	FNAG01000000
* Dokdonella koreensis* DS-123 (T)	4.45	99.2	0.56	199.6x	1	–	70.3	3557	6+47	NZ_CP015249
* Dyella japonica* DSM 16301 (T)	4.24	84.98	1.59	35x	217	114 180	64.1	3758	3+44	JPLA01000000
* Frateuria aurantia* DSM 6220 (T)	63.4	98.68	0.79	30x	1	–	63.4	3172	12+49	CP003350
*** Fulvimonas soli* LMG19981** (T)	**3.74**	**96.56**	**1.29**	**189.6x**	**223**	38 253	**71.6**	**3356**	**3+** **47**	**MSZV00000000**
* Luteibacter rhizovicinus* DSM 16549 (T)	4.76	99.66	1.08	–	1	–	64.7	4284	6+51	CP017480
*** Pseudofulvimonas gallinarii* DSM 21944** (T)	**3.48**	**100**	**1.6**	**293x**	**127**	99 644	**67.5**	2911	**4+** **44**	**MWQP00000000**
*** Rhodanobacter lindaniclasticus* DSM 17932** (T)	**4.06**	**99.31**	**0.72**	**213x**	**109**	94 198	**67.6**	3646	**3+** **47**	**MWIO00000000**
* Rudaea cellulosilytica* DSM 22992 (T)	4.34	99.54	1.34	–	7	1 056 321	63.7	3688	3+46	ARJQ01000000

CDS, coding DNA sequences;rRNA, Ribosomal ribonucleic acid;tRNA, Transfer ribonucleic acid.

## Introduction

In the advanced era of whole-genome sequencing, taxonomy is also advancing by providing us simpler and highly accurate alternatives to the cumbersome traditional methods. Taxonomy of the order *Xanthomonadales* [1] is highly controversial, which is now designated as *Lysobacterales* [2] based on conserved signature inserts/deletions (CSIs) [3]. Prior studies suggested the existence of some potential INDELs in the species of the known order *Xanthomonadales *. Key conserved proteins such as DNA ligase NAD-Dependent, MutS, RecA and DNA polymerase III alpha sub-unit with some amino acid insertion [3, 4] were used in the creation of new order *Lysobacterales* [3].

According to Rule 51b [1] of the list of prokaryotic names with standing in nomenclature (LPSN), (www.bacterio.net/index.html) the family name *Xanthomonadaceae* [1] is illegitimate as it contains the genus *Lysobacter* [2], which is the type genus of the family *Lysobacteraceae* [2]. In accordance with the latest classification based on conserved signature inserts/deletions (CSIs), the order *Lysobacterales* is sub-divided into two major families *Lysobacteraceae* and *Rhodanobacteraceae* [3]. Therefore, some of the *Xanthomonadales* genera were out of the order *Lysobacterales* and were kept as unassigned family and order. *Lysobacterales* are considered early divergent of bacteria across class gamma-proteobacteria [4–6]. The genus of this group falls under a wide range of phytopathogens, environmental pathogens and opportunistic human pathogens, causing various plant and human nosocomial infections [1]. Members of genus *Lysobacter* are Gram-negative, non-flagellated, gliding, flexing and aerobic [2]. The order *Lysobacterales* includes bacteria phenotypically, metabolically and ecologically diverse photoheterotrophs and chemoorganotrophs [2, 7, 8]. These are strictly aerobic or facultative anaerobic, facultative fermentative organisms and facultative methylotrophs. These are majorly aquatic, few are denitrifying, and many of them require NaCl for growth [3]. Members of the order were reported to be very diverse with potential biotechnological applications such as in the textile industry, the waste treatment industry [9], biocontrol agent against grapevine yellows [10] and high oil displacement efficiency [11]. The current taxonomy of the order *Lysobacterales* is based on phylogenomic and molecular markers to differentiate genera of previously known order *Xanthomonadales *. Genera such as *Metallibacterium* [12], *Panacagrimonas* [13], *Pseudoxanthomonas* [14], *Thermomonas* [15], *Fulvimonas* [16], *Pseudofulvimonas* [17], *Rhodanobacter* [7] were not involved in any such previous genome-based investigations to assign the accurate and robust phylogeny of the family.

In the current study, we have included genomes of the type strains available along with the six sequenced in the present study. Since this set of genomes represents the whole-genome sequence of the species in the order, this can depict the robust phylogenetic relationship across the genus of order *Lysobacterales *. We have generated high-quality draft genomes of six type species of family *Lysobacteraceae* and *Rhodanobacteraceae ,* which could be an invaluable resource for the future phylogenomic and comparative genomics study of the order.

## Methods

### Bacterial strains and culture conditions

Bacterial cultures were procured from The Leibniz Institute DSMZ - German Collection of Microorganisms and Cell Cultures GmbH and from The Belgian Co-ordinated Collections of Micro-organisms/Laboratory of Microbiology Gent Bacteria Collection (BCCM/LMG). All the isolates were grown as per media and conditions recommended by the respective culture collection centres.

### Genome sequencing, assembly and annotation

Genomic DNA extraction was performed using ZR Fungal/Bacterial DNA MiniPrep Kit (Zymo Research, Irvine, CA, USA) and quantified using Qubit 2.0 Fluorometer (Life Technologies). An Illumina sequencing library of genomic DNA was prepared using Nextera XT sample preparation kit (Illumina, San Diego, CA, USA) with dual indexing adapters. The Illumina sequencing library was sequenced using an in-house Illumina MiSeq (Illumina, San Diego, CA, USA) platform using 250*2 v2 paired-end sequencing kits in accordance to the manufacturer’s recommendations. Raw reads were automatically de-multiplexing by using the internal control software of Illumina. Assembly of the reads was performed using CLC Genomics Workbench v9.4.2 (CLC bio, Cambridge, MA, USA) with default parameters except minimum contig length set at 500 base pairs. Genome assembly quality in terms of completeness and contamination was accessed for all the sequenced genomes using CheckM v1.0.12 [18] with default parameters individually. CheckM suggest the use of genomes with at least 95% completeness and no more than 5% contamination. All the assembled genomes were submitted to NCBI and were annotated using NCBI Prokaryotic Genome Annotation Pipeline of NCBI (www.ncbi.nlm.nih.gov/genome/annotation_prok).

### Phylogenetic analysis

In total, 16S rRNA gene sequences of all the representative type strains of all the species belonging to the order *Lysobacterales* were fetched from respective International Nucleotide Sequence Database Collaboration (INSDC) number provided in LPSN. The phylogenetic tree based on the maximum-likelihood method was constructed from the multiple sequence alignment (MSA) [19] of 206 type strains of order *Xanthomonadales ,* including type strains from genus *Ignatzschineria* [20] *Wohlfahrtiimonas* [21] and genus *Vulcaniibacterium* [22] (Table S1, available in the online version of this article). MSA was performed using clustalw [23] with default parameters. *Pseudomonas aeruginosa* DSM 50071^(T)^ [24] was used as an outgroup for the phylogenetic tree construction using mega v7.0.18 [25] using a time reversible model with 1000 bootstrap replications. A phylogenomic tree based on putative conserved gene sequences was constructed using PhyloPhlAn v0.99 [26]. PhyloPhlAn measures the sequence diversity of all clades, classifies genomes from deep-branching candidate divisions through closely related subspecies and improves consistency between phylogenetic and taxonomic groupings. PhyloPhlAn uses >400 proteins, which are optimized from 3737 bacterial genomes. PhyloPhlAn uses USEARCH v5.2.32 [27] to fetch a defined set of 400 proteins and uses muscle v3.8.31 [28] to perform multiple sequence alignments of concatenated protein sequences. Phylogenetic construction was performed using FastTree v2.1 [29]. It includes 92 genomes of order *Lysobacterales* with species of genus *Ignatzschineria* and *Wohlfahrtiimonas* (Table S2)*. P. aeruginosa* DSM 50071^(T)^, a member of class *Gammaproteobacteria ,* was used as an outgroup in the phylogenetic construction.

### Genome similarty assessment

The whole-genome-based comparative study at order level is a cumbersome task due to the lack of computational tools for genus and family delineation. In order to investigate the taxonomy of the order *Lysobacterales ,* we have identified two unusual groupings from phylogenetic trees. Species of genus *Rhodanobacter* was sandwiched with unusual presence of species of genera *Dyella *, *Frateuria , Luteibacter* and *Fulvimonas* forming group one (G-I). Unusual group two (G-II) includes species from *Lysobacter , Luteimonas* and *Thermomonas *. We calculated the average nucleotide identity with OrthoANI v1.2 [30]. OrthoANI performs the average nucleotide calculation (ANI) using Usearch v5.2.32. The disparities at inter-genera level were reinvestigated by determining the percentage of conserved protein using the percentage of conserved protein (POCP) [31]. POCP uses inter-genera and inter-species cut-offs as 36 and 63%. The OrthoANI and POCP values' heat map was created using GENE-E v3.0.215 (https://software.broadinstitute.org/GENE-E/).

## Results and discussion

### Genome assembly and annotation

Raw Illumina sequencing reads were assembled to the high-quality draft genome using CLC Genomics Workbench v9.4.2. Assembled genome size remains in the range of 2.54 to 5.39 Mb, coverage in the range of 158x to 318x, N50 ranges from 38 253 to 222 295 bp. Percentage completeness and percentage contamination for each type species of the representative genus of the family has been summarized in detail with genome features and assembly statistics in Table 1. Except for *Dyella japonica* DSM 16301^(T)^ , *Lysobacter enzymogenes* ATCC 29487^(T)^ and *Metallibacterium scheffleri* DSM 24874^(T)^ all genomes were more than 98% complete and less than 2% contamination. The whole-genome sequence of *Dyella japonica* DSM 16301^(T)^ (NZ_JPLA01000000) [3] is merely 84.94% complete, and hence might bring biasness to the genome-based investigation of the genus *Dyella ,* we have dropped this genome out of our investigation.

### Phenotypic and genotypic evidence for taxonomy

Species belonging to different genera of the order *Lysobacterales* are from a very diverse range of ecology and habitat. The majority of the type species are environmental in nature except for type species of *Stenotrophomonas maltophilia* ATCC 13637^(T)^, which is a human opportunistic pathogen [32]. Species belong to a wide range of niches with diverse growth conditions and colony morphology (Table S3).

### Phylogenetic analysis

**Fig. 1.**
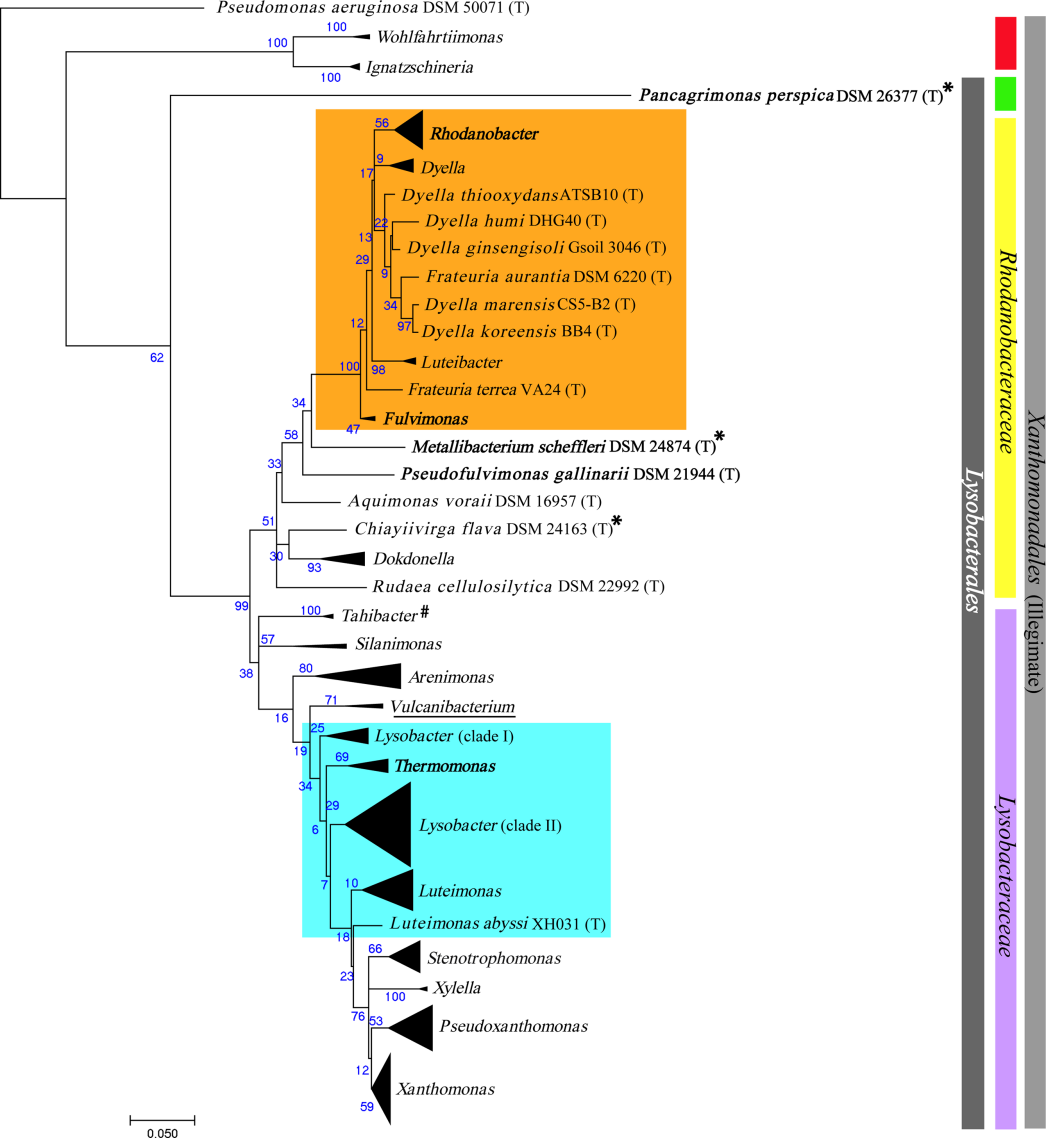
A maximum-likelihood phylogenetic tree based on 16S rRNA gene sequences of 206 type strains including 198 type strains from order *Lysobacterales *. Type species of currently unassigned genera *Wohlfahrtiimonas ,* *Ignatzschineria* (previously known members of *Xanthomonadale*) were used for phylogenetic construction. Type species superscripted with # were previously classified as *Rhodanobacteraceae* and * were defined under *Lysobacteraceae *. The tree was rooted at *Pseudomonas aeruginosa* DSM 50071^(T)^. The nodes representing genus, including multiple type strains, are clubbed and indicated with black triangles. Unusual group-I (G-I) and unusual group-II (G-II) are shaded in orange and light blue, respectively. The purple and yellow colour bar represents family *Lysobacteraceae* and *Rhodanobacteraceae ,* while grey represents the order *Xanthomonadales *. Green represents the novel family and red represents unassigned genera after revision of the order *Lysobacterales* *. Lysobacter thermophilius* YIM 77875^(T)^, which was reclassified as *Vulcaniibacterium thermophilum* forming a novel genus is underlined. The type species of the genera sequenced in this study are highlighted in bold.

The maximum-likelihood phylogenetic tree of 16S rRNA gene sequences of all 206 type strains covering all genera of the order. Here, one clade consists of type strains belonging to the family *Lysobacteraceae* and another clade of type strains of the family *Rhodanobacteraceae* (Fig. 1). Further, genus *Panacagrimonas* remain separate from the families *Lysobacteraceae* and *Rhodanobacteraceae *. The major deviations found were genera *Metallibacterium* and *Chiayiivirga ,* which are now shown to belong to the *Rhodanobacteraceae* family and were previously defined in the family *Lysobacteraceae . Lysobacter theromphilus* YIM 77875^(T)^ [33] has previously been removed from the genus *Lysobacter* and has been kept as new genus *Vulcaniibacterium* [33] out of the order *Lysobacterales *. Genus *Vulcaniibacterium* with two species falls under family *Lysobacteraceae *. Overall, from the 16S rRNA phylogenetic tree, taxonomic positioning of species of order is shuffling at the family level needs to be genomically investigated.

**Fig. 2.**
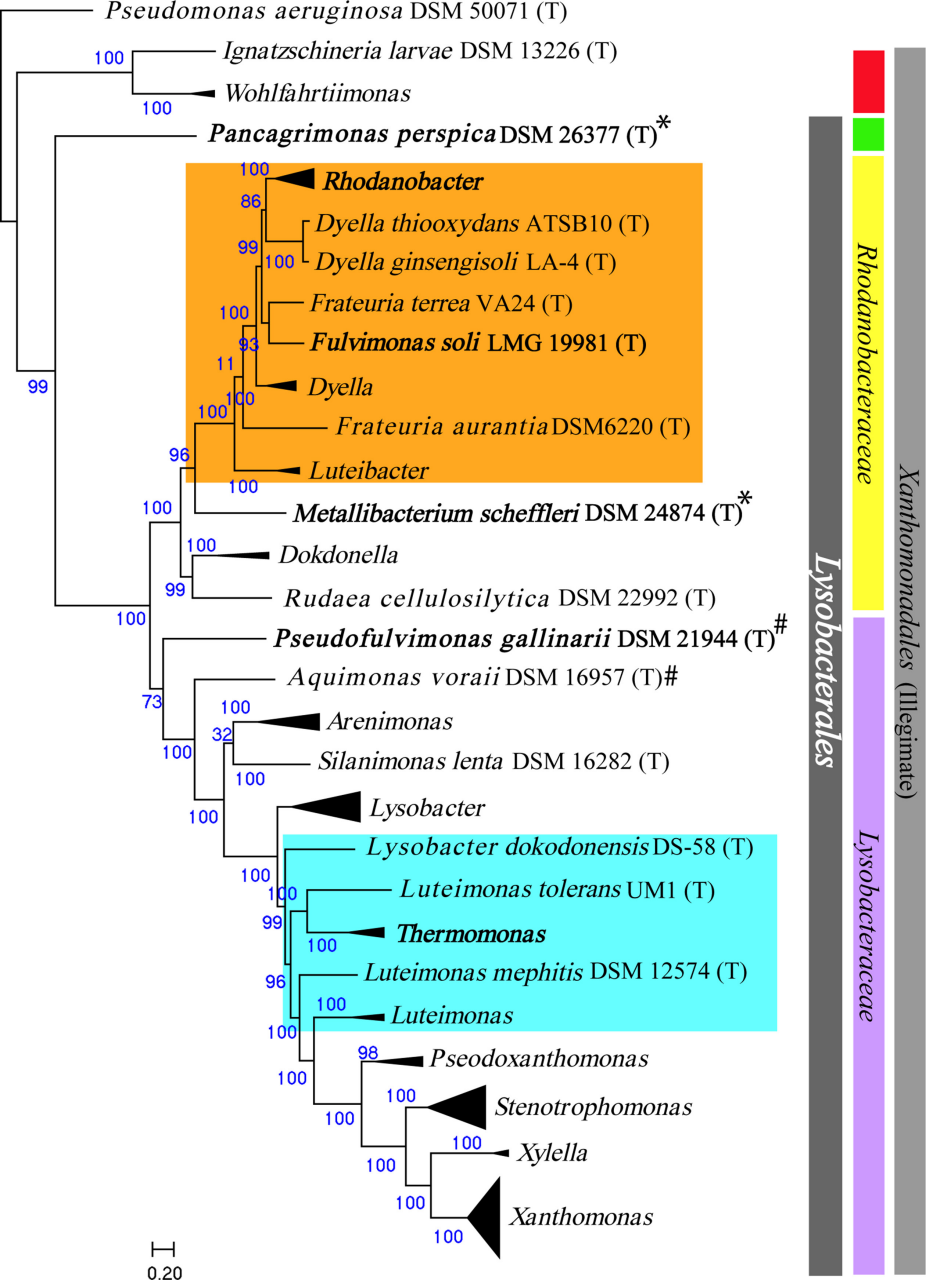
A maximum-likelihood phylogenomic tree using more than the 400 most conserved genes of all available whole-genome proteome data of the type species of order *Lysobacterales* (* Xanthomonadales *) and type species of currently unassigned genera *Wohlfahrtiimonas* and *Ignatzschineria* was included in the phylogenomic construction. *Pseudomonas aeruginosa* DSM 50071^(T)^ was used as an outgroup. The node representing genus, including multiple type strains are clubbed and indicated with black triangles. Unusual group-I (G-I) and unusual group-II (G-II) are shaded in orange and light blue, respectively. Type species superscripted with # were previously classified as *Rhodanobacteraceae* and * were defined under *Lysobacteraceae *. Genome sequences of type species are marked as bold, which are reported in the present study. The purple and yellow colour bar represents family *Lysobacteraceae* and *Rhodanobacteraceae ,* while grey represents the order *Xanthomonadales *. Green represents the novel family and red represents unassigned genera after revision of the order *Lysobacterales *.

The phylogenomic tree obtained with conserved protein sequences using PhyloPhlAn, depicts the reshuffling of genera *Aquimonas* and *Pseudofulvimonas* from family *Rhodanobacteraceae* to *Lysobacteraceae *. Further, the genus *Metallibacterium* falls in the family *Rhodanobacteraceae* and not in *Lysobacteraceae *. Genus *Panacagrimonas* remains in a separate clade (Fig. 2) to both families as seen in the case of 16S rRNA phylogenetic construction. The genera *Aquimonas *, *Pseudofulvimonas* and *Metallibacterium* contains with single species. These species were identified with the fewest closely related species with a limited method of differentiation. In addition to these, 16S rRNA and phylogenomic analysis collectively depict the presence of various misclassifications across two unusual groups with 8 (G-I) and 16 (G-II) species each. G-I holds species from genera *Rhodanobacter , Dyella , Frateuria *, *Fulvimonas* and *Luteibacter *. While G-II holds *Lysobacter dokdonensis , Thermomonas* and *Luteimonas *. Both the groups with an unusual grouping have been marked in orange and light blue in both 16S rRNA (Fig. 1) and the phylogenomic tree based on the conserved gene (Fig. 2).

### Genome similarity assessment

**Fig. 3.**
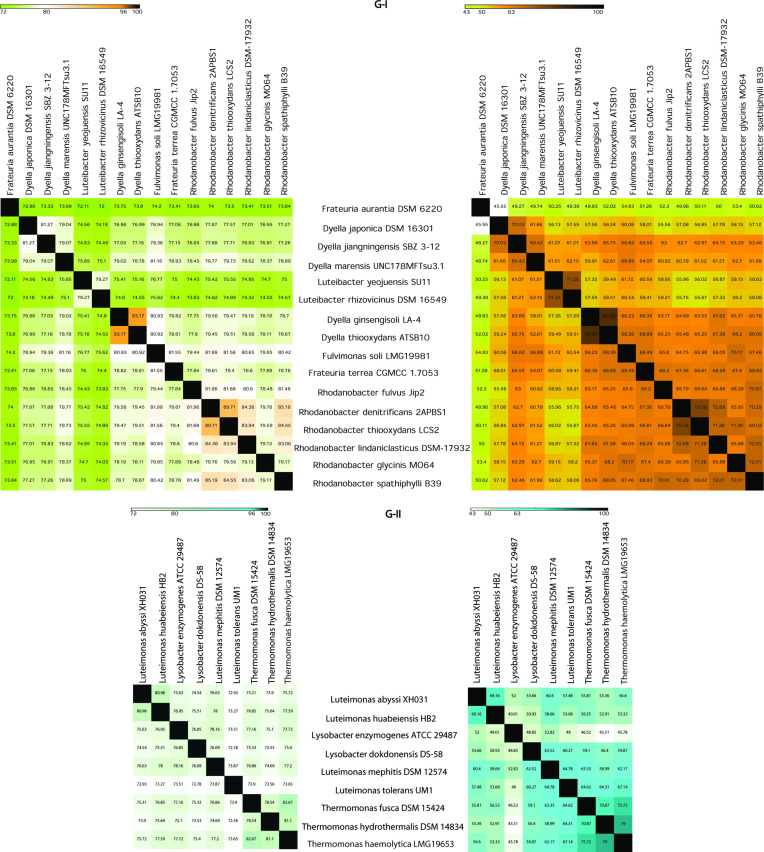
Heat map of group 1 (G-I) showing orthoANI (left) and percentage of conserved protein (POCP)[31]. POCP plot the value for *Frateuria terrea *, *Dyella thiodoxans*, *Dyella ginsengisoli* and *Fulvimonas soli* was found to be very close to species of genus *Rhodanobacter* forming putative members of the genus *Rhodanobacter *. Heat map of group 2 (G-II) showing orthoANI (left) and percentage of conserved protein (POCP)[31]. POCP value for *Luteimonas mephitis* and *Luteimonoas tolerans*, are very close to genus *Thermomonas* indicating that the species are putative members of the genus *Thermomonas *.

OrthoANI (cut-off for species boundary 96%) and POCP (cut-off for genus boundary 65%) values are in accordance with each other, reaffirming the misclassifications as obtained by phylogenetic analysis. POCP values suggest the species are diverse in nature and belong to different genera. In G-I, *Dyella ginsengisoli ,* *Dyella thiooxydans* being close to *Fulvimonas soli ,* *Frateuria terrea* forming a separate clade other than *Dyella *. Conflicts in taxonomic positioning of two species *Frateuria aurantia* and *Frateuria terrea* indicated by our phylogenomic analysis was confirmed with the POCP values (51.26) between the two species. Similarly, in G-II taxonomic position of species *Luteimonas mephitis* and *Luteimonoas tolerans* remains separate from the rest of the clade of genus *Luteimonas* in the phylogenetic tree construction obtained was confirmed with the POCP value (Fig. 3). *Luteimonas mephitis* and *Luteimonas tolerans* (POCP value of 64) are different species and possibly there is the existence of a minor unexplored genera different from rest of the *Luteimonas* clade.

Based on our findings, we suggest the reclassification of the order *Lysobacterales *. The genus *Aquimonas* and *Pseudofulvimonas* need to be removed from the family *Rhodanobacteraceae* and placed in the family *Lysobacteraceae *. The genus *Metallibacterium* and *Chiayiivirga* need to be removed from the family *Lysobacteraceae* and placed in the family *Rhodanobacteraceae *. The genus *Panacagrimonas* needs to be removed from the family *Lysobacteraceae* and kept in as an unassigned family of the order *Lysobacterales *. The genus *Vulcaniibacterium* (www.bacterio.net/vulcaniibacterium.html), which is an unassigned genus needs to be a part of family *Lysobacteraceae *. The current study is an attempt to look at the complete order *Lysobacterales* in light of whole-genome sequence information including the type species of genera belonging to the order. Such a type of whole-genome-based phylogenomics study is a prerequisite for most robust phylogeny.

## Data Bibliography

1. Definition of all valid species of the order Lysobacterales is taken from the species definition page of the LPSN List of prokaryotic names with standing in nomenclature (www.bacterio.net/-classifphyla.html) of each genera.
  The Genome (Type species) used in the phylogenomic analysis (detailed list of accession number in the Table S2).
  1. Arenimonas donghaensis DSM 18148 (T); AVCJ01000000;
  https://www.ncbi.nlm.nih.gov/nuccore/AVCJ00000000.1
  2. Luteimonas mephitis DSM 12574 (T); AULN01000000;
  https://www.ncbi.nlm.nih.gov/nuccore/AULN00000000.1
  3. Lysobacter enzymogenes ATCC 29487 (T); FNOG01000000;
  https://www.ncbi.nlm.nih.gov/nuccore/FNOG00000000.1
  4. Silanimonas lenta DSM 16282 (T); AUBD01000000;
  https://www.ncbi.nlm.nih.gov/nuccore/AUBD00000000.1
  5. Stenotrophomonas maltophilia ATCC13637 (T; CP008838;
  https://www.ncbi.nlm.nih.gov/nuccore/CP008838.1
  6. Xanthomonas campestris ATCC 33913 (T); AE008922;
  https://www.ncbi.nlm.nih.gov/nuccore/AE008922.1
  7. Xylella fastidiosa ATCC 35871 (T); AUAJ01000000;
  https://www.ncbi.nlm.nih.gov/nuccore/AUAJ00000000.1
  8. Aquimonas voraii DSM 16957 (T); FNAG01000000;
  https://www.ncbi.nlm.nih.gov/nuccore/FNAG00000000.1
  9. Dokdonella koreensis DS-123 (T); NZ_CP015249;
  https://www.ncbi.nlm.nih.gov/nuccore/CP015249.1
  10. Dyella japonica DSM 16301 (T); JPLA01000000;
  https://www.ncbi.nlm.nih.gov/nuccore/JPLA00000000.1
  11. Frateuria aurantia DSM 6220 (T); CP003350;
  https://www.ncbi.nlm.nih.gov/nuccore/CP003350.1
  12. Luteibacter rhizovicinus DSM 16549 (T); CP017480;
  https://www.ncbi.nlm.nih.gov/nuccore/CP017480.1
  13. Rudaea cellulosilytica DSM 22992 (T); ARJQ01000000;
  https://www.ncbi.nlm.nih.gov/nuccore/ARJQ00000000.1

## Supplementary Data

Supplementary material 1
