# Supplementary material for: Phylogenomics insights into order and families of Lysobacterales
Source: Access Microbiol. 2019 Apr 17;1(2):e000015. doi: 10.1099/acmi.0.000015 (PMC7470346; doi:10.1099/acmi.0.000015)
Supplement: Supplementary material 1 [file acmi-1-015-s001.pdf]

Supplementary Table S1: Accession Number type strains of species used in 16S rRNA Phylogenetic tree construction.

Sl. No.	Strain name	ACCESSION No.
1	*Aquimonas voraii* GPTSA 20	AY544768.1
2	*Arenimonas aestuarii* S2-21	KT619125.2
3	*Arenimonas daechungensis* CH15-1	JN033774.1
4	*Arenimonas daejeonensis* T7-07T	AM229325.1
5	*Arenimonas donghaensis* HO3-R19	DQ411038.1
6	*Arenimonas malthae* CC-JY-1	DQ239766.1
7	*Arenimonas maotaiensis* YT8	KF746926.1
8	*Arenimonas metalli* CF5-1	HQ698842.1
9	*Arenimonas oryziterrae* YC6267	EU376961.1
10	*Arenimonas subflava* PYM3-14T	HE616177.1
11	*Arenimonas taoyuanensis* YN2-31A	KC237721.1
12	*Aspromonas composti* TR7-09T	AM229324.1
13	*Chiayiivirga flava* DSM24163	GQ281768.1
14	*Dokdonella fugitiva* type A3T	AJ969432.1
15	*Dokdonella ginsengisoli* Gsoil 191	AB245362.1
16	*Dokdonella immobilis* LM2-5	FJ455531.1
17	*Dokdonella koreensis* DS-123	AY987368.1
18	*Dokdonella kunshanensis* DC-3	JQ341904.1
19	*Dokdonella soli* KIS28-6	EU685334.2
20	*Dyella ginsengisoli* Gsoil 3046	AB245367.1
21	*Dyella humi* DHG40	KP938953.1
22	*Dyella japonica* XD53	AB110498.1
23	*Dyella jejuensis* JP1	KF709700.2
24	*Dyella jiangningensis* SBZ3-12	HQ236051.1
25	*Dyella koreensis* BB4	AY884571.1
26	*Dyella kyungheensis* THG-B117	JX566987.1
27	*Dyella marensis* CS5-B2T	AM939778.1
28	*Dyella soli* JS12-10	EU604272.1
29	*Dyella terrae* JS14-6	EU604273.1
30	*Dyella thiooxydans* ATSB10	EF397574.1
31	*Dyella yeojuensis* R2A16-10	DQ181549.1
32	*Frateuria aurantia* IFO3245	AB091194.1
33	*Frateuria terrea* VA24	EU682683.1
34	*Fulvimonas soli* LMG 19981T	AJ311653.1
35	*Fulvimonas yonginensis* 5GHs31-2	KJ490635.1
36	*Ignatzschineria indica* FFA1	EU008088.2
37	*Ignatzschineria ureiclastica* FFA3	EU008089.2
38	*Luteibacter anthropi* CCUG 25036T	FM212561.1
39	*Luteibactor rhizovicina* LJ96T	AJ580498.1

40	*Luteimonas abyssi* XH031	KC986351.1
41	*Luteimonas aestuarii* B9	EF660758.1
42	*Luteimonas aquatica* RIB1-20	EF626688.1
43	*Luteimonas arsenica* 26-35	KP208749.2
44	*Luteimonas composti* CC-YY255	DQ846687.1
45	*Luteimonas cucumeris* Y4	HQ874629.3
46	*Luteimonas huabeiensis* HB2	JX658136.1
47	*Luteimonas lutimaris* G3	GU199001.2
48	*Luteimonas marina* FR1330	EU295459.1
49	*Luteimonas mephitis* B1953/27.1	AJ012228.1
50	*Luteimonas notoginsengisoli* SYP-B804	KP076295.1
51	*Luteimonas padinae* CDR-SL15	LN879436.1
52	*Luteimonas pelagia* CC-VAM-7	KC169811.1
53	*Luteimonas soli* Y2	KP684142.1
54	*Luteimonas terrae* THG-MD21	KJ769177.2
55	*Luteimonas terricola* BZ92r	FJ948107.1
56	*Luteimonas tolerans* UM1	KM888877.1
57	*Luteimonas vadosa* JCM 18392	AB704915.1
58	*Lysobacter aestuarii* S2-C	KT583751.2
59	*Lysobacter agri* THG-SKA3	KM576858.1
60	*Lysobacter antibioticus* ATCC 29479	AB019582.1
61	*Lysobacter arseniciresistens* ZS79	HQ315827.1
62	*Lysobacter brunescens* ATCC 29482	AB161360.1
63	*Lysobacter bugurensis* ZLD-29	EU780693.1
64	*Lysobacter caeni* BUT-8	KJ008918.1
65	*Lysobacter capsici* YC5194	EF488749.1
66	*Lysobacter cavernae* YIM C01544	KT306825.2
67	*Lysobacter concretionis* Ko07	AB161359.1
68	*Lysobacter daejeonensis* GH1-9	DQ191178.1
69	*Lysobacter defluvii* IMMIB APB-9T	AM283465.1
70	*Lysobacter dokdonensis* DS-58	EF100698.1
71	*Lysobacter enzymogenes* DSM 2043T	AJ298291.1
72	*Lysobacter erysipheiresistens* RS-LYSO-3	KT001243.1
73	*Lysobacter firmicutimachus* PB-6250	KU593484.1
74	*Lysobacter fragariae* THG-DN8.7	KM035978.1
75	*Lysobacter ginsengisoli* Gsoil 357	AB245363.1
76	*Lysobacter gummosus* ATCC 29489	AB161361.1
77	*Lysobacter hankyongensis* KTce-2	JQ349049.1
78	*Lysobacter humi* FJY8	KR698371.1
79	*Lysobacter koreensis* KCTC 12204	AB166878.1
80	*Lysobacter korlensis* ZLD-17	EU908051.1
81	*Lysobacter lycopersici* CC-Bw-6	KC820654.1
82	*Lysobacter maris* KMU-14	LC021525.1
83	*Lysobacter mobilis* 9NM-14	JQ608331.2

84	*Lysobacter niabensis* GH34-4	DQ462461.1
85	*Lysobacter niastensis* GH41-7	DQ462462.1
86	*Lysobacter novalis* THG-PC7	KM658501.1
87	*Lysobacter oligotrophicus* JCM 18257	AB694977.1
88	*Lysobacter oryzae* YC6269	EU376963.1
89	*Lysobacter panacisoli* CJ29	JQ806748.1
90	*Lysobacter panaciterrae* Gsoil 068	AB245359.1
91	*Lysobacter rhizophilus* THG-YS3.6	KT962171.1
92	*Lysobacter rhizosphaerae* THG-DN8.3	KM035979.1
93	*Lysobacter ruishenii* CTN-1	GU086401.1
94	*Lysobacter solanacearum* T20R-70	KU379668.1
95	*Lysobacter sediminicola* 7C-9	JQ349048.1
96	*Lysobacter soli* DCY21T	EF623862.1
97	*Lysobacter spongiicola* KMM 329	AB299978.2
98	*Lysobacter terrae* THG-A13	KF483861.1
99	*Lysobacter terricola* 5GH18-14	KR869779.1
100	*Lysobacter thermophilus* YIM 77875	JQ746036.1
101	*Lysobacter ximonensis* XM415	EU237492.1
102	*Lysobacter xinjiangensis* RCML-52	EU833988.1
103	*Lysobacter yangpyeongensis* GH19-3	DQ191179.1
104	*Metallibacterium scheffleri* DKE6	HQ909259.1
105	*Panacagrimonas perspica* Gsoil 142	AB257720.1
106	*Pseudofulvimonas gallinarii* Sa15T	FN298515.1
107	*Pseudomonas aeruginosa* DSM 50071T	HE978271.1
108	*Pseudomonas pictorum* LMG 981	AJ131116.1
109	*Pseudoxanthomonas broegbernensis* B1616/1	AJ012231.1
110	*Pseudoxanthomonas daejeonensis* TR6-08	AY550264.1
111	*Pseudoxanthomonas gei* ZFJR-3	KF387628.1
112	*Pseudoxanthomonas helianthi* roo10	AB905612.1
113	*Pseudoxanthomonas indica* P15	EF424397.1
114	*Pseudoxanthomonas japonensis* 12-3	AB008507.1
115	*Pseudoxanthomonas jiangsuensis* wax	NR_132712.1
116	*Pseudoxanthomonas kalamensis* JA40	AY686710.1
117	*Pseudoxanthomonas kaohsiungensis* J36	AY650027.1
118	*Pseudoxanthomonas koreensis* T7-09	AY550263.1
119	*Pseudoxanthomonas mexicana* AMX 26B	AF273082.1
120	*Pseudoxanthomonas putridarboris* WD12	GU908487.2
121	*Pseudoxanthomonas sacheonensis* BD-c54	EF575564.2
122	*Pseudoxanthomonas sangjuensis* 5GH38-5	KJ607172.1
123	*Pseudoxanthomonas spadix* IMMIB AFH-5	AM418384.1
124	*Pseudoxanthomonas suwonensis* 4M1	AY927994.1
125	*Pseudoxanthomonas taiwanensis* CB-226	AF427039.2
126	*Pseudoxanthomonas wuyuanensis* XC21-2	JN247803.1
127	*Pseudoxanthomonas yeongjuensis* GR12-1	DQ438977.1

128	*Rhodanobacter aciditrophus* sjH1	KP126755.1
129	*Rhodanobacter caeni* MJ01	GQ250431.2
130	*Rhodanobacter denitrificans* 2APBS1	FJ851443.1
131	*Rhodanobacter fulvus* JCM 21488	AB100608.1
132	*Rhodanobacter ginsengisoli* GR17-7	EF166075.1
133	*Rhodanobacter ginsenosidimutans* Gsoil 6054	EU332826.1
134	*Rhodanobacter glycinis* MO64	EU912469.1
135	*Rhodanobacter humi* RS22	KX674374.1
136	*Rhodanobacter koreensis* THG-DD7	KF532124.1
137	*Rhodanobacter lindanoclasticus* RP5557	AF039167.1
138	*Rhodanobacter panaciterrae* LnR5-47	EU332829.1
139	*Rhodanobacter rhizosphaerae* CR164	FJ772032.2
140	*Rhodanobacter soli* DCY45	FJ605268.1
141	*Rhodanobacter spathiphylli* B39	AM087226.1
142	*Rhodanobacter terrae* GP18-1	EF166076.1
143	*Rhodanobacter thiooxydans* LCS2	AB286179.1
144	*Rhodanobacter umsongensis* GR24-2	FJ821731.1
145	*Rudaea cellulosilytica* KIS3-4	EU741687.1
146	*Schineria larvae* L1/68	AJ252143.1
147	*Silanimonas lenta* 25-4	AY557615.1
148	*Silanimonas mangrovi* AK13	HE573746.1
149	*Stenotrophomonas acidaminiphila*	AF273080.1
150	*Stenotrophomonas chelatiphaga* LPM-5	EU573216.1
151	*Stenotrophomonas daejeonensis* MJ03	GQ241320.1
152	*Stenotrophomonas dokdonensis* DS-16	DQ178977.1
153	*Stenotrophomonas ginsengisoli* DCY01	DQ109037.1
154	*Stenotrophomonas humi* R-32729T	AM403587.1
155	*Stenotrophomonas koreensis* JCM 13256	AB166885.1
156	*Stenotrophomonas maltophilia* IAM 12423	AB294553.1
157	*Stenotrophomonas maltophilia* MGB	U62646.1
158	*Stenotrophomonas nitritireducens* L2	AJ012229.1
159	*Stenotrophomonas pavanii* ICB 89	FJ748683.2
160	*Stenotrophomonas rhizophila* e-p10	AJ293463.1
161	*Stenotrophomonas terrae* R-32768T	AM403589.2
162	*Stenotrophomonas tumulicola* T5916-2-1b	LC066089.1
163	*Tahibacter aquaticus* PYM5-11T	AM981201.1
164	*Tahibacter caeni* BUT-6	KJ179841.1
165	*Thermomonas brevis* LMG 21746T	AJ519989.1
166	*Thermomonas carbonis* GZ436	KF923805.1
167	*Thermomonas fusca* LMG 21737T	AJ519986.1
168	*Thermomonas haemolytica* A50-7-3	AJ300185.1
169	*Thermomonas hydrothermalis* ATCC BAA-470	AF542054.1
170	*Thermomonas koreensis* Ko06	DQ154906.1
171	*Vulcaniibacterium tengchongense* YIM 77520	JX964994.1

172	*Wohlfahrtiimonas chitiniclastica* S5	AM397063.1
173	*Wohlfahrtiimonas larvae* KBL006	JN873914.1
174	*Xanthomonas albilineans* ATCC 33915	X95918.1
175	*Xanthomonas alfalfae citrumelonis* LMG 9325	NR_104965.1
176	*Xanthomonas alfalfae* LMG 495	NR_104957.1
177	*Xanthomonas arboricola* ATCC 49083	Y10757.1
178	*Xanthomonas axonopodis* ATCC 19312	X95919.1
179	*Xanthomonas bromi* CFBP 1976	Y10764.1
180	*Xanthomonas campestris* ATCC 33913	AE008922
181	*Xanthomonas campestris pv. phaseoli* ATCC 49119	GU993265.1
182	*Xanthomonas cassavae* ICMP 204	Y10762.1
183	*Xanthomonas citri* ATCC 49118	NR_104964.1
184	*Xanthomonas citri malvacearum* DSM 3849	NR_117146.1
185	*Xanthomonas codiaei* ATCC 700187	Y10765.1
186	*Xanthomonas cucurbitae* CFBP 2542	Y10760.1
187	*Xanthomonas cynarae* CFBP 4188	AF208315.1
188	*Xanthomonas dyei* ICMP 12167	NR_104949.1
189	*Xanthomonas euvesicatoria* DSM19128T	FR733718.1
190	*Xanthomonas fragariae* ATCC 33239	X95920.1
191	*Xanthomonas fuscans* LMG 826	NR_104958.1
192	*Xanthomonas gardneri* DSM 19127T	FR749911.1
193	*Xanthomonas hortorum* ICMP 453	Y10759.1
194	*Xanthomonas hyacinthi* ATCC 19314	Y10754.1
195	*Xanthomonas maliensis* M97	KF992843.1
196	*Xanthomonas melonis* ICMP 8682	Y10756.1
197	*Xanthomonas oryzae* Dye YK9	X95921.1
198	*Xanthomonas perforans* DSM 18975T	FR749910.1
199	*Xanthomonas pisi* ATCC 35936	Y10758.1
200	*Xanthomonas populi* ATCC 51165	X95922.1
201	*Xanthomonas sacchari* CFBP 4641	Y10766.1
202	*Xanthomonas theicola* ATCC 700184	Y10763.1
203	*Xanthomonas vasicola* LMG 736	Y10755.1
204	*Xanthomonas vesicatoria* ATCC 35937	Y10761.1
205	*Xylella fastidiosa* PL.788	AF203388.1
206	*Xylella fastidiosa* ATCC 35879	AF192343.1

Supplementary Table S2: Accession Number for the type strains used in construction of whole genome phylogeny using PhyloPhlAn.

Sl. No	Strain Name	Accession Number
1	*Aquimonas voraii* DSM 16957(T)	FNAG01000001
2	*Arenimonas composti* DSM 18010(T)	NZ_AUFF01000001
3	*Arenimonas donghaensis* DSM18148(T)	NZ_AVCJ01000001
4	*Arenimonas malthae* CC-JY-1(T)	NZ_AVCH01000001
5	*Arenimonas metalli* CF5-1(T)	NZ_AVCK01000001
6	*Arenimonas oryziterrae* DSM 21050(T)	NZ_ATVD01000001
7	*Dokdonella immobilis* CGMCC 1.7659(T)	FOVF01000001
8	*Dokdonella koreensis* DS-123(T)	NZ_CP015249
9	*Dyella ginsengisoli* LA-4(T)	NZ_AMSF01000001
10	*Dyella japonica* DSM 16301(T)	NZ_CP008884
11	*Dyella jiangningensis* SBZ 3-12(T)	NZ_CP007444
12	*Dyella marensis* UNC178MFTsu3.1(T)	FONH01000001
13	*Dyella thiooxydans* ATSB10(T)	NZ_CP014841
14	*Frateuria aurantia* DSM 6220(T)	NC_017033
15	*Frateuria terrea* CGMCC 1.7053(T)	FNYC01000018
16	*Fulvimonas soli* LMG 19981(T)	**MSZV00000000**
17	*Ignatzschineria larvae* DSM 13226(T)	NZ_AZOD01000001
18	*Luteibacter rhizovicinus* DSM 16549(T)	NZ_CP017480
19	*Luteibacter yeojuensis* SU11(T)	NZ_JZRB01000001
20	*Luteimonas abyssi* XH031(T)	NZ_KQ759763
21	*Luteimonas huabeiensis* HB2(T)	NZ_JAAN01000001
22	*Luteimonas mephitis* DSM 12574(T)	NZ_KE383816
23	*Luteimonas tolerans* UM1(T)	NZ_FTLW01000020
24	*Lysobacter antibioticus* ATCC 29479(T)	NZ_CP013141
25	*Lysobacter arseniciresistens* ZS79(T)	NZ_AVPT01000001
26	*Lysobacter capsici* 55(T)	NZ_CP011130
27	*Lysobacter concretionis* Ko07 DSM 16239(T)	NZ_AVPS01000001
28	*Lysobacter daejeonensis* GH1-9(T)	NZ_AVPU01000007
29	*Lysobacter defluvii* IMMIB APB-9 DSM 18482(T)	NZ_AUHT01000004
30	*Lysobacter dokdonensis* DS-58(T)	NZ_JRKJ01000001
31	*Lysobacter enzymogenes* ATCC 29487(T)	NZ_FNOG01000023
32	*Lysobacter gummosus* 3.2.11(T)	NZ_CP011131
33	*Lysobacter spongiicola* DSM 21749(T)	NZ_FUXP01000001
34	*Metallibacterium scheffleri* DSM-24874(T)	**MWQO00000000**
35	*Panacagrimonas perspica* DSM-26377(T)	**MWIN00000000**
36	*Pseudofulvimonas gallinarii* DSM-21944(T)	**MWQP00000000**
37	*Pseudomonas aeruginosa* DSM50071(T)	NZ_JYLC01000010
38	*Pseudoxanthomonas dokdonensis* DSM 21858(T)	NZ_LDJL01000001
39	*Pseudoxanthomonas indica* P15(T)	NZ_FUZV01000002
40	*Pseudoxanthomonas wuyuanensis* CGMCC 1.10978(T)	OCND01000001
41	*Rhodanobacter denitrificans* 2APBS1(T)	NC_020541
42	*Rhodanobacter fulvus* Jip2(T)	NZ_AJXU01000001

43	*Rhodanobacter glycinis* MO64(T)	FOSR01000001
44	*Rhodanobacter lindaniclasticus* DSM-17932(T)	**MWIO00000000**
45	*Rhodanobacter spathiphylli* B39(T)	NZ_AJXT01000001
46	*Rhodanobacter thiooxydans* LCS2(T)	NZ_AJXW01000001
47	*Rudaea cellulosilytica* DSM 22992(T)	NZ_KB899241
48	*Silanimonas lenta* DSM 16282(T)	NZ_AUBD01000001
49	*Stenotrophomonas acidaminiphila* JCM 13310(T)	NZ_CP012900
50	*Stenotrophomonas chelatiphaga* DSM 21508(T)	NZ_LDJK01000001
51	*Stenotrophomonas daejeonensis* JCM 16244(T)	NZ_LDJP01000001
52	*Stenotrophomonas ginsengisoli* DSM 24757(T)	NZ_LDJM01000001
53	*Stenotrophomonas humi* DSM 18929(T)	NZ_LDJI01000001
54	*Stenotrophomonas koreensis* DSM 17805(T)	NZ_LDJH01000001
55	*Stenotrophomonas africana* LMG 22072(T)	NZ_LLXW01000001
56	*Stenotrophomonas maltophilia* ATCC 13637(T)	NZ_MTGD01000001
57	*Stenotrophomonas pictorium* JCM 9942(T)	NZ_BAZI01000001
58	*Stenotrophomonas nitritireducens* DSM 12575(T)	NZ_CP016756
59	*Stenotrophomonas pavanii* DSM 25135(T)	NZ_LDJN01000001
60	*Stenotrophomonas rhizophila* QL-P4(T)	NZ_CP007597
61	*Stenotrophomonas terrae* DSM 18941(T)	NZ_LDJJ01000001
62	*Thermomonas fusca* DSM 15424(T)	NZ_AUIV01000001
63	*Thermomonas hydrothermalis* DSM 14834(T)	NZ_FQUK01000085
64	*Thermomonas haemolytica* LMG19653(T)	**MSZW00000000**
65	*Wohlfahrtiimonas chitiniclastica* DSM 18708(T)	NZ_AQXD01000001
66	*Wohlfahrtiimonas larvae* KBL006(T)	NZ_MVDO01000001
67	*Xanthomonas alfalfae* alfalfae LMG495(T)	JPYG01000001
68	*Xanthomonas euvesicatoria* pv. citrumelonis strain CFBP3371	NZ_MDCC01000001
69	*Xanthomonas arboricola* pv. Jugalandis CFBP 2528(T)	NZ_JZEF01000001
70	*Xanthomonas axonopodis* pv. axonopodis LMG 982(T)	NZ_JPYE01000001
71	*Xanthomonas bromi* LMG 947(T)	NZ_FLTX01000134
72	*Xanthomonas campestris* ATCC 33913(T)	NC_003902
73	*Xanthomonas cannabis* NCPPB 3753(T)	NZ_JSZF01000001
74	*Xanthomonas cassavae* CFBP 4642(T)	NZ_CM002139
75	*Xanthomonas citri* LMG9322(T)	NZ_JPYD01000087
76	*Xanthomonas euvesicatoria* LMG 27970(T)	NZ_JPYC01000009
77	*Xanthomonas floridensis* WHRI 8848 LXNG01.1(T)	LXNG01000001
78	*Xanthomonas fragariae* PD8851(T)	NZ_LT853882
79	*Xanthomonas fuscans* pv. aurantifolii ICPB 11122(T)	ACPX01000001
80	*Xanthomonas fuscans* NCPPB 381(T)	NZ_JTKK02000049
81	*Xanthomonas gardneri* ATCC 19865(T)	NZ_AEQX01000206
82	*Xanthomonas hortorum* pv. carotae M081(T)	NZ_CM002307
83	*Xanthomonas maliensis* M97(T)	NZ_AQPR01000001
84	*Xanthomonas nasturtii* WHRI 8853(T)	NZ_LYMI01000001
85	*Xanthomonas oryzae* ATCC 35933(T)	NZ_KI519402
86	*Xanthomonas perforans* CFBP 7293(T)	NZ_MOLQ01000001
87	*Xanthomonas phaseoli* pv. dieffenbachiae LMG 695(T)	NZ_CP014347
88	*Xanthomonas pisi* DSM 18956(T)	NZ_JPLE01000001
89	*Xanthomonas vasicola* NCPPB 2417(T)	NZ_JSBW02000055

90	*Xanthomonas vesicatoria* ATCC 35937(T)	NZ_CP018725
91	*Xylella fastidiosa* ATCC 35879(T)	NZ_JQAP01000001
92	*Xylella taiwanensis* PLS229(T)	NZ_JDSQ01000001

Supplementary table S3: Phenotypic feature of the type species of the order *Lysobacterales.*

Type Species	Nos of species	% GC	Growth Temperature	Isolation Source	Colony morphology	Motility	Reference
***Aquimonas voraii* DSM 16957 (T)**	1	68.5	38 ° C; pH 7.2	Warm spring	Circular, yellowish-brown, glistening and undulated margins	+	(1)
***Dokdonella koreensis* DS-123 (T)**	6	70.3	30 °C ; pH 6.5	Soil	Circular, convex, glistening, smooth, moderate yellow	+	(2)
***Dyella japonica* DSM 16301 (T)**	12	67.2	25–30 ° C; pH 6.5-7.2	Soil	Yellow	+	(3)
***Frateuria aurantia* DSM 6220 (T)**	2	63.4	30 ° C; pH 3.6	Liliurn auratum	Dark yellow, glistering, convex with an undulate or regular edge	+	(4)
***Fulvimonas soli* LMG 19981 (T)**	2	70.7	28 ° C; pH 7.8	Soil	Deep-yellow, mucoid, low convex, translucent	+	(5)
***Luteibacter rhizovicinus* DSM 16549 (T)**	3	64.7	25-30 ° C; pH 6-9	Rhizosphere	Yellow, low-convex	+	(6)
***Pseudofulvimonas gallinarii* DSM 21944 (T)**	1	67.5	25-30 ° C; pH	Duck barn air	Yellow, translucent and shiny	-	(7)
***Rhodanobacter lindaniclasticus* DSM 17932 (T)**	17	67.6	30 ° C; pH 7	Soil	Yellow colonies with clean edges	-	(8)
***Rudaea cellulosilytica* DSM 22992 (T)**	1	63.7	28-30 ° C; pH 6-7	Soil	Yellow, round or irregular	+	(9)
***Tahibacter aquaticus* DSM 21667 (T)**	2	65.4	20-28 ° C; pH 5-7	Drinking water	Bright yellow, translucent, convex, with entire edge	-	(10)
***Arenimonas donghaensis* DSM 18148 (T)**	11	65	28 ° C;<br>pH 7-9	Seashore sand	Yellowish white,<br>Translucent and convex	-	(11)
***Chiayiivirga flava* DSM 24163 (T)**	1	68.6	30 ° C;<br>pH 7	Agricultural soil	Round yellow pigmented	-	(12)
***Luteimonas mephitis* DSM 12574 (T)**	18	68.5	25 ° C;	Ammonia supplied biofilters	Yellow pigmented	NA	(13)
***Lysobacter enzymogenes* ATCC 29487 (T)**	45	69	25 ° C;<br>pH 5-10	Soil	Irregular slimy/mucoid and white, cream, yellow, pink or brown transparent colony	-	(14)
***Metallibacterium scheffleri* DSM 24874 (T)**	1	66.6	25- 30 °C; pH 5.5	Acidic biofilm of coper mine	Yellow pigmented	-	(15)
***Panacagrimonas perspica* DSM 26377 (T)**	1	69.9	30 ° C;<br>pH 6.5-7.0	Ginseng field	Smooth, flat, white, and irregular shapes	+	(16)
***Pseudoxanthomonas broegbernensis* DSM 12573 (T)**	19	70.7	25 ° C	Experimental biofilter	Yellow pigmented	NA	(13)
***Silanimonas lenta* DSM 16282 (T)**	2	71.1	47 ° C pH 9	Hot spring	Pale yellow, translucent, irregular and sticky	+	(17)
***Stenotrophomonas maltophilia* ATCC13637 (T)**	14	66.3	28 ° C	Pleural fluid	Smooth, glistening, with an entire margin, white, greyish, or pale yellow	+	(18)
***Thermomonas haemolytica* LMG 19653 (T)**	6	67.1	37-50 ° C	Kaolin slurry	Whitish/translucent, circular, smooth and convex with an entire edge	+	(19)
***Xanthomonas campestris* ATCC 33913 (T)**	32	65.1	° C pH	Na crucifers	Yellow mucoid smooth	+	(20)
***Xylella fastidiosa* ATCC 35871 (T)**	2	52	27 ° C pH 6.5	Pierce's disease of grapevines	Convex to pulvinate smooth opalescent with entire margins and umbonate rough with finely undulated margins	-	(21)

References

1. Saha P, Krishnamurthi S, Mayilraj S, Prasad GS, Bora TC, Chakrabarti T. Aquimonas voraii gen. nov., sp. nov., a nov
el
gammaproteobacterium isolated from a warm spring of Assam, India. International Journal of Systematic and Evolutionary Microbiology. 2005;55(4):1491-5.
2. Yoon J-H, Kang S-J, Oh T-K. Dokdonella koreensis gen. nov., sp. nov., isolated from soil. International Journal of Systematic and Evolutionary Microbiology. 2006;56(1):145-50.
3. Xie C-H, Yokota A. Dyella japonica gen. nov., sp. nov., a γ-proteobacterium isolated from soil. International Journal of Systematic and Evolutionary Microbiology. 2005;55(2):753-6.
4. SWINGS J, GILLIS M, KERSTERS K, DE VOS P, GOSSELÉ F, DE LEY J. Frateuria, a New Genus for "Acetobacter aurantius". International Journal of Systematic and Evolutionary Microbiology. 1980;30(3):547-56.
5. Mergaert J, Cnockaert MC, Swings J. Fulvimonas soli gen. nov., sp. nov., a gamma-proteobacterium isolated from soil after enrichment on acetylated starch plastic. International Journal of Systematic and Evolutionary Microbiology. 2002;52(4):1285-9.
6. Johansen JE, Binnerup SJ, Kroer N, Mølbak L. Luteibacter rhizovicinus gen. nov., sp. nov., a yellow-pigmented gammaproteobacterium isolated from the rhizosphere of barley (Hordeum vulgare L.). International Journal of Systematic and Evolutionary Microbiology. 2005;55(6):2285-91.
7. Kämpfer P, Martin E, Lodders N, Langer S, Schumann P, Jäckel U, et al. Pseudofulvimonas gallinarii gen. nov., sp. nov., a new member of the family Xanthomonadaceae. International Journal of Systematic and Evolutionary Microbiology. 2010;60(6):1427-31.
8. Nalin R, Simonet P, Vogel TM, Normand P. Rhodanobacter lindaniclasticus gen. nov., sp. nov., a lindane-degrading bacterium. International Journal of Systematic and Evolutionary Microbiology. 1999;49(1):19-23.
9. Weon H-Y, Yoo S-H, Kim Y-J, Lee C-M, Kim B-Y, Jeon Y-A, et al. Rudaea cellulosilytica gen. nov., sp. nov., isolated from soil. International Journal of Systematic and Evolutionary Microbiology. 2009;59(9):2308-12.
10. Makk J, Homonnay ZG, Kéki Z, Lejtovicz Z, Márialigeti K, Spröer C, et al. Tahibacter aquaticus gen. nov., sp. nov., a new gammaproteobacterium isolated from the drinking water supply system of Budapest (Hungary). Systematic and applied microbiology. 2011;34(2):110-5.
11. Kwon S-W, Kim B-Y, Weon H-Y, Baek Y-K, Go S-J. Arenimonas donghaensis gen. nov., sp. nov., isolated from seashore sand. International Journal of Systematic and Evolutionary Microbiology. 2007;57(5):954-8.
12. Hsu Y-H, Lai W-A, Lin S-Y, Hameed A, Shahina M, Shen F-T, et al. Chiayiivirga flava gen. nov., sp. nov., a novel bacterium of the family Xanthomonadaceae isolated from an agricultural soil, and emended description of the genus Dokdonella. International Journal of Systematic and Evolutionary Microbiology. 2013;63(9):3293-300.
13. Finkmann W, Altendorf K, Stackebrandt E, Lipski A. Characterization of N2O-producing Xanthomonas-like isolates from biofilters as Stenotrophomonas nitritireducens sp. nov., Luteimonas mephitis gen. nov., sp. nov. and Pseudoxanthomonas broegbernensis gen. nov., sp. nov. International journal of systematic and evolutionary microbiology. 2000;50(1):273-82.
14. CHRISTENSEN P, Cook F. Lysobacter, a new genus of nonfruiting, gliding bacteria with a high base ratio. International Journal of Systematic and Evolutionary Microbiology. 1978;28(3):367-93.
15. Ziegler S, Waidner B, Itoh T, Schumann P, Spring S, Gescher J. Metallibacterium scheffleri gen. nov., sp. nov., an alkalinizing gammaproteobacterium isolated from an acidic biofilm. International Journal of Systematic and Evolutionary Microbiology. 2013;63(4):1499-504.
16. Im W-T, Liu Q-M, Yang J-E, Kim M-S, Kim S-Y, Lee S-T, et al. Panacagrimonas perspica gen. nov., sp. nov., a novel member of Gammaproteobacteria isolated from soil of a ginseng field. The Journal of Microbiology. 2010;48(2):262-6.
17. Lee EM, Jeon CO, Choi I, Chang K-S, Kim C-J. Silanimonas lenta gen. nov., sp. nov., a slightly thermophilic and alkaliphilic gammaproteobacterium isolated from a hot spring. International Journal of Systematic and Evolutionary Microbiology. 2005;55(1):385-9.
18. Palleroni NJ, Bradbury JFJIJoS, Microbiology E. Stenotrophomonas, a new bacterial genus for Xanthomonas maltophilia (Hugh 1980) Swings et al. 1983. 1993;43(3):606-9.
19. Busse HJ, Kämpfer P, Moore ERB, Nuutinen J, Tsitko IV, Denner EBM, et al. Thermomonas haemolytica gen. nov., sp. nov., a gamma-proteobacterium from kaolin slurry. International Journal of Systematic and Evolutionary Microbiology. 2002;52(2):473-83.
20. Dowson D. On the systematic position and generic names of the Gram negative bacterial plant pathogens. Zentralblatt fur Bakteriologie, Parasitenkunde und Infektionskrankheiten, 2. 1939;100:177-93.

21. Wells JM, Raju BC, Hung H-Y, Weisburg WG, Mandelco-Paul L, Brenner DJ. Xylella fastidiosa gen. nov., sp. nov: gram-negative, xylem-limited, fastidious plant bacteria related to Xanthomonas spp. International Journal of Systematic and Evolutionary Microbiology. 1987;37(2):136-43.
